# Decreased Expression of Vitamin D Receptor Affects an Immune Response in Primary Biliary Cholangitis via the VDR-miRNA155-SOCS1 Pathway

**DOI:** 10.3390/ijms18020289

**Published:** 2017-01-29

**Authors:** Agnieszka Kempinska-Podhorodecka, Malgorzata Milkiewicz, Urszula Wasik, Joanna Ligocka, Michał Zawadzki, Marek Krawczyk, Piotr Milkiewicz

**Affiliations:** 1 Department of Medical Biology, Pomeranian Medical University, 70-111 Szczecin, Poland; agnieszkakempinska@interia.eu (A.K.-P.); wasikula@gmail.com (U.W.); 2 Department of General Transplant and Liver Surgery, Medical University of Warsaw, 02-097 Warszawa, Poland; jligocka@gmail.com (J.L.); michu244@interia.pl (M.Z.); marek.krawczyk@wum.edu.pl (M.K.); 3 Translation Medicine Group, Pomeranian Medical University, 70-111 Szczecin, Poland; p.milkiewicz@wp.pl; 4 Liver and Internal Medicine Unit, Medical University of Warsaw, 02-097 Warszawa, Poland

**Keywords:** vitamin D receptor, suppressor of cytokine signaling 1 (SOCS1), miR-155, cholestatic liver disease

## Abstract

Primary biliary cholangitis (PBC) is an immune-mediated cholestatic disease. Vitamin D receptor (VDR)-dependent signaling constrains an inflammatory response by targeting the miRNA155-SOCS1 (suppressor of cytokine signaling 1) axis. The VDR-miRNA155-SOCS1 pathway was investigated in the context of the autoimmune response associated with PBC. Human liver tissues from non-cirrhotic PBC (*n* = 22), cirrhotic PBC (*n* = 22), cirrhotic primary sclerosing cholangitis (PSC, *n* = 13), controls (*n* = 23), and peripheral blood mononuclear cells (PBMC) obtained from PBC (*n* = 16) and PSC (*n* = 10) patients and healthy subjects (*n* = 11) were used for molecular analyses. VDR mRNA and protein expressions were substantially reduced in PBC livers (51% and 59%, respectively). Correspondingly, the decrease of SOCS1 protein expression in PBC livers, after normalization to a marker of lymphocytes and forkhead family transcriptional regulator box P3 (FOXP3, marker of Treg), was observed, and this phenomenon was accompanied by enhanced miRNA155 expression. In PSC livers, protein expressions of VDR and SOCS1 were comparable to the controls. However, in PBM cells, protein expressions of VDR and SOCS1 were considerably decreased in both PBC and PSC. We demonstrated that VDR/miRNA155-modulated SOCS1 expression is decreased in PBC which may lead to insufficient negative regulation of cytokine signaling. These findings suggest that the decreased VDR signaling in PBC could be of importance in the pathogenesis of PBC.

## 1. Introduction

Primary biliary cholangitis (PBC) is characterized by the immune-mediated destruction of small intrahepatic bile ducts and portal inflammation that may lead to the progressive development of liver fibrosis and liver failure [1]. Even though the pathogenesis of PBC is not entirely understood, immunological features are believed to play an important role. The presence of distinctive anti-mitochondrial antibodies (AMAs), the serological hallmark of PBC, and autoreactive T and B cells was shown to participate in liver inflammation [2,3]. Adaptive autoimmunity and cholangiocyte apoptosis is increased in PBC by over-activated Th1, Th2, and Th17 cells as a result of decreased immunosuppressive functions of regulatory T cells (Tregs), which are essential for maintaining immune homeostasis [4,5,6].

The hormonal form of Vitamin D has physiological effects that are much broader than the maintenance of mineral homeostasis, and basic, genetic, and epidemiological studies indicate a potential role of vitamin D in the prevention of autoimmune diseases. Vitamin D is a well-known immune modulator of both innate and adaptive immunities. It acts by inhibition of T cell activation and proliferation, suppression of pro-inflammatory cytokines, or induction of differentiation of forkhead family transcriptional regulator box P3 (FOXP3)-positive regulatory T cells [7,8,9]. These vitamin D–dependent modulations essentially lessen autoreactivity. Biological effects of vitamin D are mediated through the vitamin D receptor (VDR), an NR1I family receptor with transcription factor activities, which forms a heterodimer with Retinoid X receptor (RXR) and binds to DNA response elements in target genes [10]. VDR is ubiquitously expressed and a widespread function for VDR has been recognized [11]. There are growing data on the contribution of vitamin D deficiency to autoimmunity [12,13].

Non-coding microRNAs (miRNAs) are a class of naturally occurring short RNAs of about 22 nucleotides that regulate the post-transcriptional expression of genes by either degradation of target mRNA transcripts, or by repression of mRNA translation [14]. A new research area has recently emerged, focusing on the role of individual miRNA in lymphocyte biology. Among various microRNAs, miR155, a miRNA largely restricted to hematopoietic cells, is of particular interest. It is up-regulated upon lymphocyte activation to control cell proliferation and differentiation [15]. Furthermore, miRNA-155 shapes cytokine signaling via down-regulation of the suppressor of cytokine signaling (SOCS) proteins, which are involved in the inhibition of inflammation [16]. Members of the SOCS family play an essential role in immunological homeostasis [17]. SOCS1-knockout mice died within three weeks of birth due to over-activation of peripheral T cells and necrosis of the liver [18], whereas T cell–specific SOCS1-conditional knockout (cKO) mice developed autoimmune inflammatory diseases [19,20]. Moreover, polymorphism of SOCS1 has been associated with PBC [21].

Of late, a novel regulatory mechanism for vitamin D to control innate immunity has been identified. It was demonstrated that vitamin D/VDR signaling diminishes inflammation by augmenting the negative feedback regulation by targeting the miR-155-SOCS1 pathway [22]. Knowing the importance of vitamin D in maintaining a tolerogenic dendritic cell phenotype, the evaluation of VDR expression may lead to better understanding of the pathogenesis of autoimmune liver diseases. Until now, the vast majority of research related to vitamin D signaling in PBC was focused on genetic studies and demonstrated correlations between VDR polymorphism and clinical presentation or the presence of a more progressive form of PBC [23,24]. In the past, expression of VDR was demonstrated in primary human hepatocytes and biliary epithelial cells as well as in liver tissue of patients with chronic liver disease [25,26]. However, the data on VDR expression in livers of cholestatic patients are very limited. Similarly, little is known about the efficiency of anti-inflammatory mechanisms in immune-mediated chronic cholestatic liver conditions occurring in PBC and primary sclerosing cholangitis (PSC). In the present study we attempted to clarify a function of the VDR-regulated signaling pathway in liver tissue and peripheral blood mononuclear cells (PBMC) of patients with PBC and PSC.

## 2. Results

### 2.1. Liver

**Figure 1:**
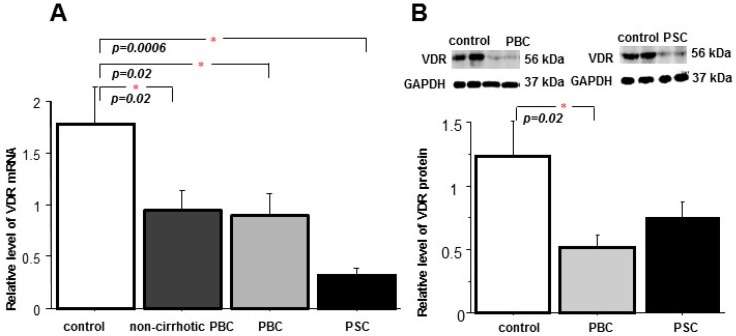
Expression of VDR in liver tissue. (**A**) *VDR* gene expression was significantly reduced both in non-cirrhotic (*n* = 22) and cirrhotic (*n* = 22) livers patients of primary sclerosing cholangitis (PBC) as well as in cirrhotic livers patients of primary sclerosing cholangitis (PSC, *n* = 13). Levels of mRNA expression were normalized with eukaryotic 18S rRNA Endogenous Control and presented as a fold-change relative to control; (**B**) VDR protein expression was substantially decreased in cirrhotic liver tissue of patients with PBC (*n* = 22) when compared to control tissue (*n* = 23), but not in cirrhotic PSC (*n* = 13). Changes in protein levels were determined by densitometry analyses after normalization to GAPDH (glyceraldehyde-3-phosphate dehydrogenase) as a control for loading. Bars indicate the mean ± SEM (standard error of the mean).

**Figure 2:**
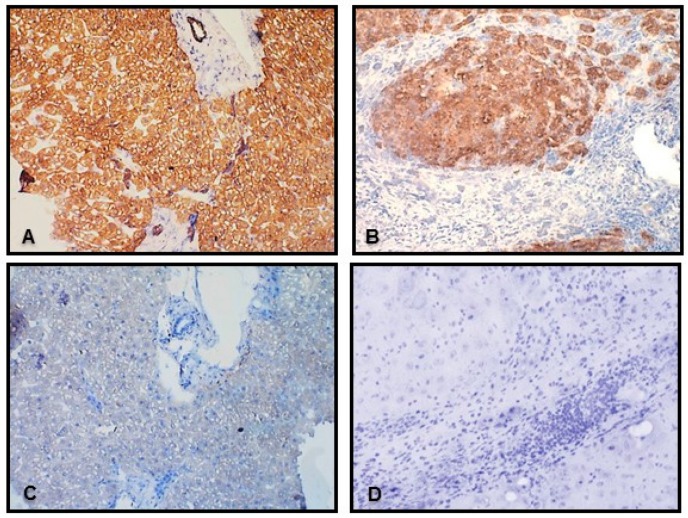

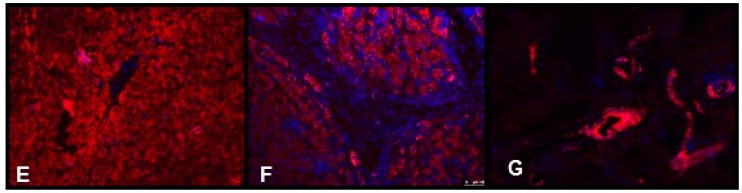
Immunohistochemistry for VDR in livers of patients with cirrhotic stage of PBC and healthy controls. Representative photomicrographs of human liver sections stained with anti-VDR antibody in controls (**A**,**B**), in nodules of cirrhotic PBC (**B**,**F**) and fibrous septa containing bile ducts in PSC (**G**). VDR protein are depicted by brown (light micrographs: **A**,**B**) or red staining (immunofluorescence: **E**–**G**), whereas nuclei are stained blue with hematoxylin (**A**–**D**) or DAPI (**E**,**F**). Negative controls without incubation with primary antibody are presented for control (**C**) and PBC (**D**). Original magnifications: × 200; or × 400 (**G**).

Expression of the *VDR* gene was substantially suppressed in livers of non-cirrhotic and cirrhotic patients with PBC when compared to controls (53% reduction, *p* = 0.02, and 51% reduction *p* = 0.02, respectively). Similarly, VDR mRNA levels were decreased in cirrhotic livers of PSC patients (0.5 ± 0.2 in PSC vs. 1.6 ± 0.4 in controls, *p* = 0.0006, Figure 1A). Correspondingly, the VDR protein level was considerably reduced in the cirrhotic PBC or PSC group (0.5 ± 0.3 in PBC vs. 1.2 ± 0.3 in controls, *p* = 0.02) (Figure 1B). However, the reduction of VDR protein expression was much smaller in PSC and did not reach statistical significance (0.6 ± 0.1 in PSC vs. 1.2 ± 0.3 in controls)) (Figure 1B). Immunohistochemistry depicted VDR protein in all hepatocytes and in cholangiocytes of biliary ducts, but not in periduct fibrotic tissue or septal connective tissue (Figure 2). Accordingly, the VDR protein is evenly distributed across normal hepatic parenchyma and bile ductules (Figure 2A,E), whereas in cirrhotic livers of both PBC and PCS patients, the presence of VDR proteins was limited to nodular areas (Figure 2B,F) and irregular ductular structures at the edge of the nodules (Figure 2B,G).

**Figure 3:**
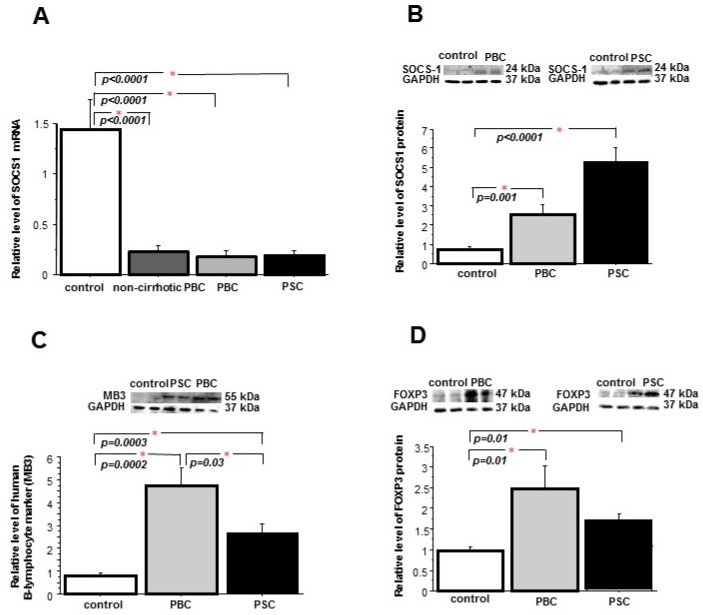

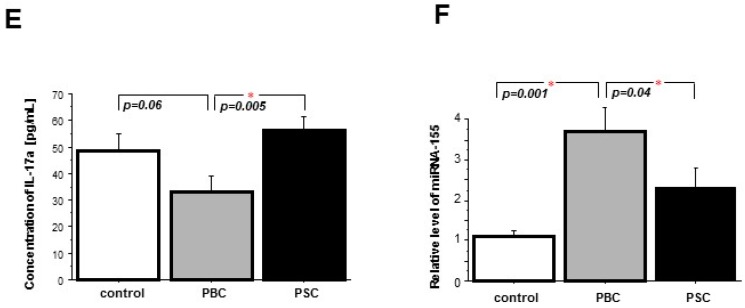
Expression of suppressor of cytokine signaling 1 (SOCS1), lymphocyte marker (MB3), forkhead family transcriptional regulator box P3 (FOXP3), interleukin-17A (IL-17A) and miRNA-155 in liver tissue. (**A**) SOCS1 mRNA expression was significantly reduced both in non-cirrhotic and cirrhotic livers of PBC (*n* = 22 and *n* = 22, respectively) and PSC patients (*n* = 13). Levels of mRNA were presented as a fold-change relative to controls after normalization to 18S rRNA Endogenous Control (Hs99999901_s1, Applied Biosystems, Foster City, CA, USA); (**B**) SOCS1 protein levels were substantially increased in cirrhotic PBC (*n* = 22), and PSC (*n* = 13) which were accompanied by enhanced expression of (**C**) lymphocyte marker (MB3; sc-52340, Santa Cruz Biotechnology, Santa Cruz, CA, USA); and (**D**) FOXP3 protein. Protein levels were determined by densitometry analyses after normalization to GAPDH as a control for loading; (**E**) Hepatic concentration of IL-17A was substantially increased in PSC in comparison to PBC; (**F**) Enhanced expression of miR-155 was observed in livers of patients with PBC in comparison to controls and PSC. RNU44 (001091) served as reference microRNA. (**C**,**D**) Results are representative of *n* = 13–19 independent experiments per group. Bars indicate the mean ± SEM.

Gene expression of the VDR signaling target, SOCS1, was greatly decreased in non-cirrhotic PBC and in cirrhotic PBC and PSC (*p* < 0.0001, *p* < 0.0001 and *p* < 0.0001 vs. controls, respectively) (Figure 3A). However, SOCS1 protein levels were substantially increased in livers of PBC (3.4-fold; *p* = 0.001 vs. controls) and PSC patients (7.5-fold; *p* < 0.0001 vs. controls) (Figure 3B). A significant positive correlation was observed between the expression of SOCS1 mRNA and VDR protein (Table 1).

Knowing that liver tissues of patients with PBC and PSC are characterized by enlarged numbers of infiltrating leukocytes producing SOCS1 protein, we evaluated the level of lymphocyte marker in hepatic tissue. We observed a significant increase of this marker level both in PBC and PSC (*p* = 0.0002 and *p* = 0.0003 vs. controls, respectively) (Figure 3C) but the augmentation of leukocyte infiltration in PSC was significantly smaller than in PBC (*p* = 0.03). The analysis of FOXP3 expression, the marker of regulatory T cells, confirmed the increased infiltration of T-reg in PBC (2.5-fold, *p* = 0.01 vs. controls) and PSC (1.7-fold, *p* = 0.01 vs. controls) (Figure 3D). There was a tendency (*p* = 0.06) in the reduction of the hepatic concentration of IL-17A, a signature cytokine for Th17 in PBC (33.1 ± 6.1 pg/mL in PBC vs. 48 ± 6.1 pg/mL in controls). In contrast, the concentrations of IL-17A in PSC livers were increased and significantly higher than in PBC (*p* = 0.005 vs. PBC) (Figure 3E). Since it was reported that SOCS1-deficient naive CD4 + T cells were poorly differentiated into Th17 as a result of STAT3 suppression [19], we evaluated the expression of the active form of STAT3. In our study the concentration of phospho-STAT3 protein was significantly lower in liver tissue of PBC patients (133.6 ±11.8 pg/µL in controls vs. 47.4 ± 8.6 pg/µL in PBC; *p* < 0.0001). As a growing number of miRNAs have emerged as regulators of immune response [27], and it has been postulated that vitamin D down-regulates inflammation via targeting miRNAs, we evaluated the level of miR-155. The expression of miR-155 was significantly increased in PBC livers in comparison to either controls (3.7-fold, *p* = 0.001) or PSC livers (*p* = 0.04) (Figure 3F). 

A significant negative correlation was observed between the expression of miR155 and VDR mRNA (Table 1). Notably, the miR155 level also correlated significantly with the SOCS1 protein level (Table 1), which may be interpreted as an indication of the miR155-mediated epigenetic regulation of SOCS1 expression within PBC livers.

**Figure 4:**
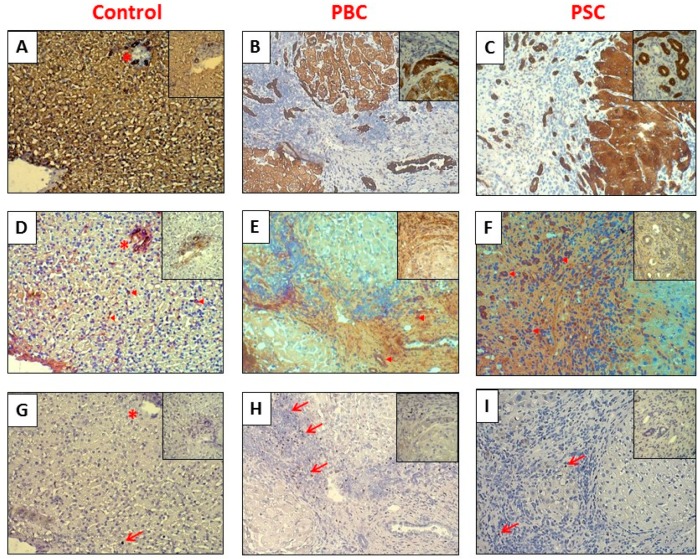
Representative immunohistochemical findings of VDR, SOCS1, and FOXP3 proteins in serial sections of liver tissue from healthy controls (**A**,**D**,**G**), cirrhotic PBC (**B**,**E**,**H**), and PSC patients (**C**,**F**,**I**). In healthy controls, SOCS1 proteins (**D**) were located in portal tracts (asterisks) and biliary ducts within hepatic parenchyma (arrowhead). In cirrhotic liver tissue of PBC (**E**) and PSC patients (**F**), SOCS1 proteins were mainly located within fibrous septa and irregular bile ductules (arrowhead). Regulatory T cells co-expressing FOXP3 (arrows) were hardly found in healthy controls (**G**), whereas cirrhotic livers of PBC (**H**) and PSC (**I**) contained a high number of FOXP3+ T cells within fibrotic tissue (arrows). Original magnifications 200×. Inserts (400×) contain the representative images of either a portal tract in healthy tissue (**A**,**D**,**G**) or a nodule margin with surrounding fibrotic tissue in PBC (**B**,**E**,**H**) and PSC (**C**,**F**,**I**).

The enhanced expressions of SOCS1 and FOXP3 proteins were visualized by immunostaining (Figure 4). In healthy hepatic tissue, SOCS1 was mainly located in portal tracts and bile ductules (Figure 4D), whereas FOXP3 proteins were hardly detected and only single FOXP3-positive cells were present in the analyzed field (Figure 4G). However, in cirrhotic livers of both PBC and PSC patients, SOCS1 proteins were mainly limited to the area of fibrotic tissue surrounding nodules and irregular ductular structures (Figure 4E,F), while numerous FOXP3-positive cells were identified in a thick sleeve of dense fibrous tissue and in abnormal portal tracks (Figure 4H,I).

### 2.2. Peripheral Blood Mononuclear Cells (PBMCs)

**Figure 5:**
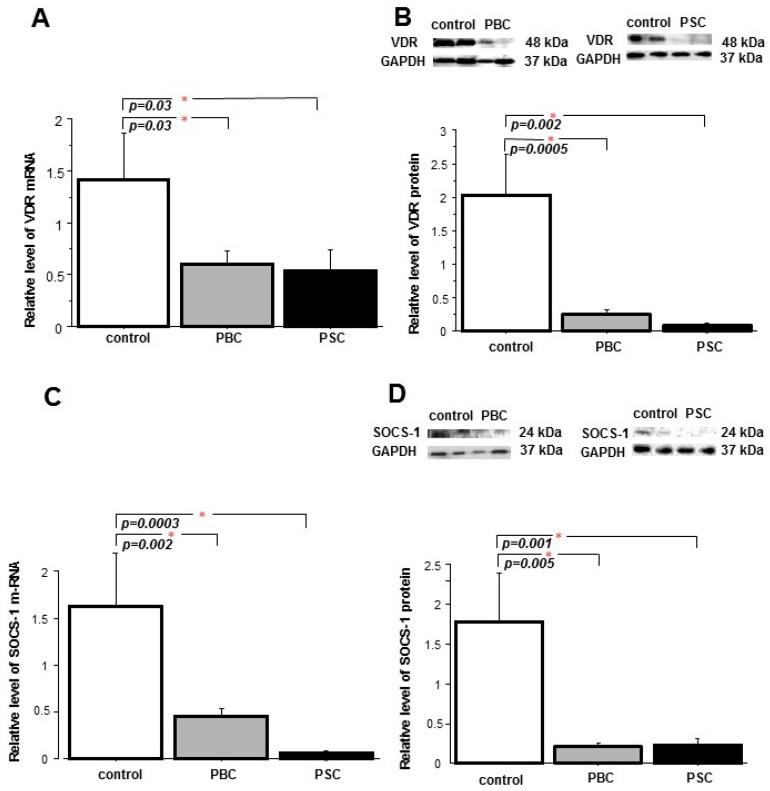
Expression of VDR, SOCS1 in peripheral blood mononuclear cells of PBC and PSC patients. The decreased level of VDR mRNA (**A**) and protein levels (**B**) were observed in PBM cells of patients with PBC and PSC, which was accompanied by the reduction of *SOCS1* gene (**C**) and SOCS1 protein (**D**) expressions. Levels of mRNA were presented as a fold-change relative to controls after normalization to 18S rRNA expression. Protein levels were assessed by Western blot, and quantified relative to GAPDH. The number of experiments per group: PBC *n* = 16, PSC *n* = 10, control *n* = 11. Bars indicate the mean ± SEM.

**Figure 6:**
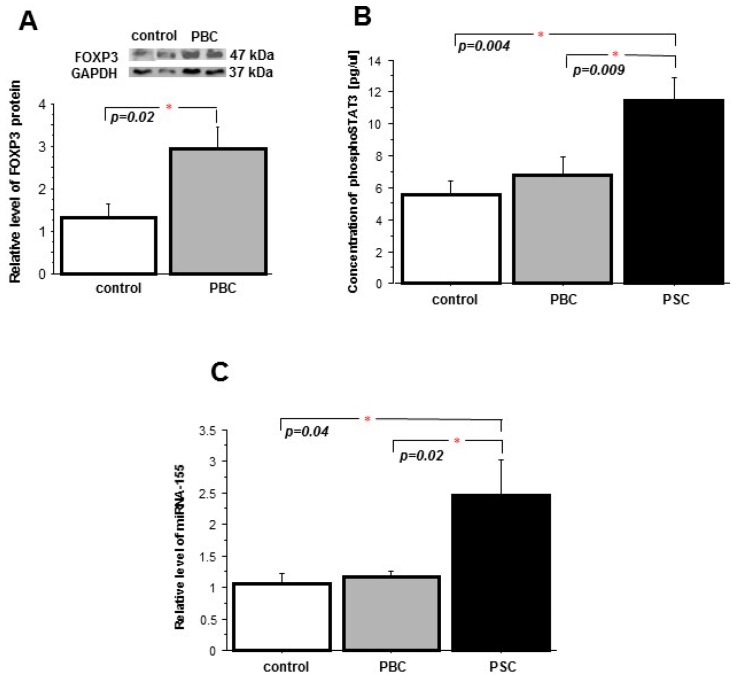
Expression of FOXP3, miR-155 and phospho-STAT3 in peripheral blood mononuclear cells of PBC and PSC patients. Increased level of FOXP3 protein (**A**) suggests the enlargement of Treg subpopulation in PBM cells of patients with PBC. In comparison to controls, concentration of phospho-STAT3 [pY705] (Multispecies ELISA Kit; ThermoFisher Scientific, Carlsbad, CA, USA) (**B**) and level of miR-155 (477927_mir; miR-191 served as reference microRNA; Applied Biosystems) were enhanced in PBM cells of PSC patients (**C**). The number of experiments per group: PBC *n* = 16, PSC *n* = 10, control *n* = 11. Bars indicate the mean ± SEM.

We sought to examine whether the changes observed in cholestatic PBC and PSC livers are present in peripheral blood mononuclear cells of patients with PBC. The mRNA and protein levels of VDR were markedly lower in PBMCs of both PBC patients (57% and 80% reduction, *p* = 0.026 and *p* = 0.0005 vs. controls, respectively,) and PSC patients (62% and 90% reduction, *p* = 0.027 and *p* = 0.002 vs. controls, respectively) (Figure 5A,B). Comparably, the expressions of the *SOCS1* gene and the SOCS1 protein were significantly suppressed in both PBC (0.5 ± 0.08 vs. 1.6 ± 0.6, *p* = 0.002 vs. controls, and 0.2 ± 0.04 vs. 1.8 ± 0.6; *p* = 0.005 vs. controls) and PSC (0.06 ± 0.01 vs. 1.6 ± 0.6, *p* = 0.0003 vs. controls, and 0.3 ± 0.02 vs. 1.8 ± 0.6; *p* = 0.001 vs. controls, respectively) (Figure 5C,D). In addition, the FOXP3 protein level was significantly higher in the PBMC of PBC patients in comparison to the controls (2.9 ± 0.5 vs. 1.3 ± 0.3, *p* = 0.02) (Figure 6A). The concentration of phospho-STAT3 was considerably increased in PBM cells of PSC patients (*p* = 0.004 vs. controls and *p* = 0.009 vs. PBC) (Figure 6B) and, similarly, in the PBMC of PSC patients there was a significant enhancement of miR-155 expression (*p* = 0.04 vs. controls and *p* = 0.02 vs. PBC) (Figure 6C).

## 3. Discussion

**Figure 7:**
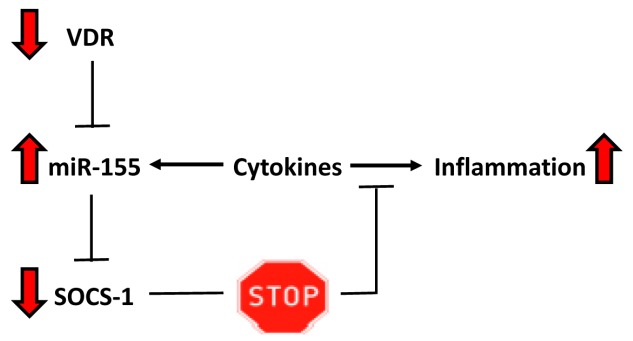
Schematic representation of the inadequate VDR signaling in primary biliary cholangitis (PBC). Vitamin D–VDR signaling limits the inflammatory response by targeting the miR-155-SOCS1 pathway. We postulate that, as a result of the reduction of VDR expression, the negative feedback inhibition loop is not maintained and inflammatory response is sustained in PBC. This is caused by miR-155 up-regulation leading to excessive suppression of SOCS1. Red arrows depict the changes observed in liver tissue of patients with PBC.

The present study provides novel insight into pathological changes present in human liver tissue of patients with PBC and PSC, which may account for inadequate VDR signaling and impaired suppression of inflammatory reactions. We observed a substantial reduction of VDR mRNA and protein levels in PBMCs of patients with PBC and PSC. However, the VDR protein levels in liver tissue, as well as SOCS1 and miR155 expressions, were different in these two cholestatic conditions. In contrast to PSC, the PBC livers’ VDR protein levels were significantly reduced. Moreover, the enhanced miR155 expression was accompanied by a lower synthesis of SOCS1 protein, whereas in PSC the expression of miR155 was comparable to the controls and was associated with a higher amount of SOCS1. Thus, in PBC livers the SOCS1 translation was decreased, likely impeding the negative feedback regulation of the inflammatory response (Figure 7).

Deficiency in 25-hydroxyvitamin D was found to be a feature of not only PBC, but also chronic liver diseases in general [28]. Decreased levels of vitamin D in PBC correlated with disease manifestations and co-morbidity with other autoimmune diseases [29]. It is worth mentioning that in contrast to previous reports, our groups of PBC patients, both the donors of liver tissues and PBM cells, had normal serum levels of biologically active 1,25-dihydroxyvitamin D (Table 2). Thus, in the present study, we showed that despite normal levels of 1,25(OH)_2_D_3_, the insufficient expression of VDR may be responsible for an impaired translation of vitamin D−induced signaling, which may contribute to a sustained inflammatory reaction.

In addition to VDR reduction, we have recorded a substantial suppression of the *SOCS1* gene in livers of patients with early and advanced stages of PBC and PSC. Conversely, the hepatic protein level of SOCS1 was considerably increased in both conditions. This unsuspected observation may be explained by the influx of infiltrating lymphocytes which are the main source of the SOCS1 protein. Indeed, the levels of the lymphocyte marker and FOXP3 protein (a marker of Treg cells) were substantially enhanced in cirrhotic liver tissues. Of interest, the magnitude of those changes was different in these two cholestatic conditions. In the livers of PBC patients, the relative levels of hepatic lymphocytes and Treg increased by 4.8 times and 2.5 times, respectively, in comparison to the controls, and those changes were accompanied by a 3.4-fold increase in the SOCS1 protein level. However, in PSC, an increase in SOCS1 protein was much more prominent (7.5-fold vs. control) and was 2.2 times higher than in PBC; however, the population of infiltrating lymphocytes was increased only by 2.8 times and FOXP3 protein by 1.7 times. Observed dissimilarities between these two cholestatic conditions may suggest that the production of SOCS1 protein by infiltrating lymphocytes is different in PBC (the ratio of SOCS1 to the lymphocyte marker was 0.7 in PBC vs. 1.1 in controls) compared to PSC (the ratio of SOCS1 to the lymphocyte marker was 2.6 vs. 1.1 in controls). The substantial inhibition of SOCS1 expression in livers of patients with PBC may potentially lead to aberrant tuning of the immune response and can result in sustained inflammation. 

SOCS1 is well recognized as an important negative modulator of both helper (Th) and regulatory T (Treg) cells [17]. T cell-specific SOCS1-deficient mice developed autoimmune inflammatory diseases with age, and were very sensitive to dextran sulfate sodium (DSS)-induced colitis [30] and ConA-induced hepatitis [31]. Recent studies have indicated that Th17/Treg imbalance plays a role in the pathogenesis of autoimmunity [32]. The number of Th17 cells increased significantly, whereas the Treg population decreased dramatically in the peripheral blood of patients with PBC, and these changes correlated with the enhanced expression of Th17-related cytokines [33,34]. Moreover, it was reported that an initially predominant Th1 reaction is gradually superseded by a Th17 response during the progression of PBC [5,35]. However, in our study we did not observe the enhanced production of interleukin (IL)-17A, which is a signature cytokine for Th17 cells. Our analysis of the IL-17A concentration in liver tissue of PBC patients did not show any increase in comparison to healthy controls. Moreover, in PBC livers, we observed a tendency toward the reduction of IL-17A synthesis (*p* = 0.06), while in the liver tissue of PSC patients there was an increase in the IL17A concentration. The explanation for these discrepancies may be extrapolated from experiments in which the *SOCS1* gene is deleted. Most SOCS1^−/−^ CD4-naive T cells differentiated into Th1, even under Th2 or Th17-skewing conditions, whereas Th17 differentiation was strongly suppressed [19]. Similarly, in SOCS1-deficient T cells, the expression of protein which suppresses Th17 differentiation, eomesodermin, was up-regulated under Th17-skewing conditions [36]. Our observations that IL17A is not enhanced in the livers of PBC patients go along with the report by Katt and colleagues who demonstrated that stimulation of PBMC with heat-inactivated bacteria led to significantly higher frequencies of Th17 and Th1/Th17 cells only in PSC patients but not in PBC patients or healthy controls [37]. The suppression of Th17 differentiation may also be explained by the reduced STAT3 activation [19]. Indeed, in our study the concentration of the phosphorylated form of STAT3 (pSTAT3) was not increased in the PBMC of PBC patients or even was reduced in PBC livers in comparison to the controls. In contrast, in the PBMC of PSC patients there was a significant increase in pSTAT3. 

Furthermore, SOCS1 plays a role in the expansion and function of regulatory T cells, formerly known as suppressor T cells, which are crucial for maintaining peripheral tolerance to self-antigens by preventing the proliferation and effector function of autoreactive T cells [38]. In humans, Tregs are defined by the expression of CD4, CD25, and FOXP3, which are required for their development and function [39]. SOCS1 prevents the production of inflammatory cytokines from Tregs, and in the absence of SOCS1, Tregs lose FOXP3 expression and become pathogenic T cells [40,41]. It was reported that the population of Tregs is increased in chronic viral hepatitis and in hepatocellular carcinoma [42,43], whereas Tregs are reduced in PBC [6,33,44]. In the current study we presented contradictory results. A more than two-fold increase in the FOXP3 protein level was observed not only in liver tissue which was infiltrated by lymphocytes but also in PBMC, which may suggest that the population of T cells is substantially enhanced by Treg cells. It is noteworthy that our study is consistent with a recent report demonstrating a higher number of *FoxP3+ Tregs* in the livers of patients with a variety of autoimmune liver conditions including PBC, whereas in non-diseased human livers the frequency of *FoxP3+ Tregs* was low [45]. The authors suggested that inflammation persists in the presence of so many Tregs, probably due to the suppressed function of Tregs within the liver microenvironment, seeing that only 5%–15% of the liver-infiltrating FOXP3+ cells expressed pSTAT5, the marker of intrahepatic Tregs activation [45], and the defective function of Tregs has been implicated in PBC [44].

The present study revealed an excessive repression of SOCS1 with the concomitant up-regulation of hepatic miR-155 in PBC. However, in PSC patients, we did not observe the up-regulation of miR-155 (NS vs. controls, *p* = 0.04 vs. PBC). miR-155, encoded by a gene known as *bic*, plays important immuno-modulatory roles. The baseline expression of miRNA-155 in a variety of immune cells is usually low until stimulated by antigens, Toll-like receptor ligands, or inflammatory cytokines. In activated cells, miR-155 targets SOCS1, which allows an inflammatory reaction to proceed and be sustained [22]. Moreover, miR155 maintains the competitive fitness and homeostasis of Treg cells and the up-regulation of FOXP3 promotes the expansion of Treg cells by targeting SOCS1 via miR155 [46]. In the absence of SOCS1, Tregs become harmful effector T cells that may induce severe colitis, liver degeneration, or lymphoid deficiencies [41,47]. miR-155, by targeting the SOCS1 protein, may prolong the inflammatory response by inhibition of the negative feedback regulation. Consequently, in the absence of VDR, miR-155 up-regulation may lead to deregulation of the negative feedback loop via inhibition of SOCS1, which triggers a sustained inflammatory response. 

## 4. Patients and Methods

### 4.1. Patients’ Characteristics and Tissue Specimens

Molecular analyses were carried out in both human liver tissue and peripheral blood mononuclear cells (PBMC) from different groups of patients. Firstly, cirrhotic liver tissues were obtained from patients with PBC (*n* = 22) or with primary sclerosing cholangitis (PSC, *n* = 13) with histologically proven cirrhosis who underwent liver transplantation. Additionally, non-cirrhotic patients with PBC (*n* = 22) who underwent percutaneous liver biopsy for histological assessment were included in this study. Control liver tissues (*n* = 23) were secured from large margin liver resections of colorectal metastases with no microscopic changes of liver disease identified by a pathologist [25,26]. Secondly, peripheral blood mononuclear cells (PBMC) were obtained from patients with PBC (*n* = 16), PSC (*n* = 10) and healthy subjects (*n* = 11). Serum level of 1,25-dihdroxyvitamin D (IBL International, Hamburg, Germany) was measured in a group of non-cirrhotic (*n* = 22) and cirrhotic patients (*n* = 22) with PBC, as well as in all donors of PBMC (*n* = 16) and the accepted reference range for normal adults was 48–110 pmol/L. All patients with PBC were supplemented with vitamin D/calcium and had normal levels of serum vitamin D. Table 1 summarizes clinical and laboratory features of patients included in the study. The study was approved by the institutional Ethic Committee of Pomeranian Medical University in Szczecin, Poland (BN-001/43/06; 20 April 2006). Written consent from subjects was obtained according to the Declaration of Helsinki.

Liver tissue (controls, cirrhotic PBC and PSC) was immediately frozen in liquid nitrogen and stored at −75 °C until used. Percutaneous needle liver biopsies (non-cirrhotic PBC) were either stored in RNAlater (Applied Biosystems, Carlsbad, CA, USA) or fixed in 10% formalin for histological assessment. PBMCs were isolated from venous blood samples using standard Roswell Park Memorial Institute (RPMI) solutions and Ficoll-Paque gradient separation (Pharmacia, GE Healthcare BioSciences AB, Uppsala, Sweden).

### 4.2. RNA and miRNA Expression Analysis

Total RNA was isolated using RNeasy Mini kit (Qiagen, Hilden, Germany) and cDNA synthesis was carried out using Superscript II RT kit (Invitrogen, Carlbad, CA, USA) according to the protocol previously described [48]. Expressions of specific genes were measured using human gene expression assays: VDR (Hs00172113_m1); SOCS1 (Hs00705164_s1); 18S rRNA (Hs99999901_s1)); and a 7500 Fast Real-Time PCR System (Applied Biosystems, Foster City, CA, USA). 

miR-155 cDNA synthesis was carried out using either the TaqMan^®^MicroRNA Reverse Transcription Kit or TaqMan Advanced miRNA cDNA synthesis kit (Applied Biosystems, USA) according to the manufacturer’s protocol. In liver tissue, the expression of miR-155 (002623) and reference microRNA, RNU44 (001091) were measured using TaqMan^®^ miRNA assays and TaqMan^®^ Universal PCR Master Mix No AmpErase (Applied Biosystems, USA). In PBMCs the expression of miR-155 (477927_mir) and reference microRNA, miR-191 (477952_mir) were measured using TaqMan^®^ Advanced miRNA assays and TaqMan^®^ Fast Advanced Master Mix (Applied Biosystems, USA). The fluorescence data were analyzed with 7500 Software v2.0.2. (Applied Biosystems, USA) and expressions of target genes were calculated using the ΔΔ*C*t method of relative quantification.

### 4.3. Protein Expression Analysis 

The following antibodies were used: VDR (sc-13133), SOCS1 (sc-9021), lymphocyte marker (sc-52340), GAPDH (sc-25778) (Santa Cruz Biotechnology, Santa Cruz, CA, USA), and FOXP3 (ab20034, Abcam), peroxidase conjugated anti-rabbit (Amersham, GE Heathcare-BioScieces AB, Uppsala, Sweden), and peroxidase conjugated anti-mouse (Amersham). Proteins were extracted by homogenization of tissue samples in Radioimmunoprecipitation assay buffer (RIPA buffer) containing protease inhibitor cocktail and PhosSTOP (Roche Diagnostic, Mauuheim, Germany). Proteins were electrophoresed in SDS polyacrylamide gels, blotted into polyvinylidene difluoride (PVDF) membranes under semi-dry transfer conditions (Thermo Scientific, Rockford, IL, USA), followed by exposure to primary and secondary antibodies. Bands were visualized by chemiluminescence detection (Chemiluminescent HRP Substrate, Millipore, Billerica, MA, USA) and quantified using MicroChemi 2.0 System and GelQuant software (Maale HaHamisha, Jerusalem, Israel).

Plasma and tissue concentrations of IL-17A and phospho-STAT3 [pY705] (Multispecies ELISA Kit; ThermoFisher Scientific, Carlsbad, CA, USA) were measured using a human ELISAKits (EHIL17A and KHO0481 ThermoFisher Scientific, Rochford, IL, USA).

### 4.4. Immunohistochemistry

Immunohistochemistry analyses on frozen liver sections (6 µm) were carried out according to the previously described procedure [48]. Biotinylated anti-mouse/anti-rabbit IgG (BA-1400, Vector Laboratories, Burlingame, CA, USA) served as secondary antibody and reactions were visualized using Vectastain Elite ABC (Vector Laboratories) and DAB kits (DAKO, Carpinteria, CA, USA). The negative controls were included in the study and uniformly demonstrated no reaction. Immunofluorescence localization of VDR protein was carried out with anti-VDR (sc-13133) followed by incubation with Rhodamine-conjugated anti-mouse IgG (Jackson ImmunoResearch, Burlingame, CA, USA). Vectrashield mounting medium with DAPI (4′,6-diamidyno-2-fenyloindol) were used to envision cell nuclei. All images were acquired with a ZEISS AxioImager Z2 microscope (Carl Zeiss, Microscopy GimbH, Jena, Germany) equipped with the ZenPro 2011 acquisition program (Carl Zeiss, Microscopy GimbH, Jena, Germany).

### 4.5. Statistics

The two-tailed Student’s *t*-test was used to compare two groups; multiple groups’ comparisons were performed with Fisher’s exact test or ANOVA with the StatView^®^ Program (SAS Institute Inc., Cary, NC, USA). Results were considered statistically significant when two-sided *p*-values were <0.05. Data are displayed as mean and SEM if not indicated otherwise. Correlation analyses were performed using the Spearman Rank method.

## 5. Conclusions

In light of the presented findings, we conclude that inadequate VDR signaling in primary biliary cholangitis with subsequent, miRNA155-modulated SOCS1 expression may lead to the insufficient negative feedback regulation of cytokine signaling. This phenomenon could be of importance in the pathogenesis of PBC. 

## Figures and Tables

**Table 1:** Spearman rank correlations for VDR, SOCS1 and miR155 expressions analyzed in liver tissues of patients with PBC and PSC.

Parameters		PBC		PSC	
		Rho	*p*-Value	Rho	*p*-Value
VDR protein	SOCS-1 protein	−0.252	0.147	−0.319	0.202
	SOCS-1 mRNA	0.402	**0.017**	0.319	0.154
	miR-155	0.151	0.441	0.454	0.089
VDR mRNA	SOCS-1 protein	0.069	0.697	−0.118	0.637
	SOCS-1 mRNA	0.234	0.159	0.469	**0.027**
	miR-155	0.714	**0.058**	−0.028	0.926
miR-155	SOCS-1 protein	0.694	**0.001**	0.421	0.167
	SOCS-1 mRNA	−0.344	0.169	−0.286	0.449

Value in boldface indicate statistically significant difference (Tied *p*-Value, Spearman Rank Correlation, StatView).

**Table 2:** Clinical and biochemical characteristics of patients with PBC and PSC.

Variables	Liver			Peripheral Blood Mononuclear Cells	
	PBC non-cirrhotic *n* = 22	PBC cirrhotic *n* = 22	PSC *n* = 13	PBC *n* = 16	PSC *n* = 10
Number (male/female)	0/22	1/21	8/5	1/15	14/6
Age, years	52 ± 11	56 ± 9	44 ± 14.1	52.6 ± 7.3	35.1 ± 10.8
AST IU/L	62.6 ± 56.1	147.9 ± 128.0	260. ± 348.4	83 ± 63.6	67.6 ± 33.9
ALP IU/L	265.4 ± 182.9	447.4 ± 296.4	543.7±454.3	111 ± 109.5	306.6 ± 44.9
Bilirubin (µmol/L)	21.6 ± 28.8	114.0 ± 112.4	64.8 ± 91.9	15.1 ± 9.6	85.5 ± 67.5
1,25-(OH)_2_-Vitamin D (pmol/L) 1,25-(OH)_2_-Vitamin D (range)	66.6 ± 20.5 (36.8−117.1)	85.1 ± 9.8 (67.8−114.3)	-	62.6 ± 4.7 (44.2−112.9)	-

Values are given as mean ± SD, unless stated otherwise. ALP = Alkaline Phosphatase; ALT = Alanine aminotransferase.

## Abbreviations

PBC: Primary biliary cholangitis
VDR: Vitamin D receptor
SOCS1: Suppressor of cytokine signaling1
PSC: Primary sclerosing cholangitis
PBMC: Peripheral blood mononuclear cells
AMAs: Antimitochondrial antibodies
Treg: Regulatory T cells
FOXP3: Forkhead family transcriptional regulatorbox-P3
FOXP3+ Treg: FOXP3-positive regulatory T cells
STAT3: Signal transducer and activator of transcription-3
